# Progressive Cone‐Rod Synaptic Dysfunction in Dynamin‐1 ( *DNM1* ) Related Developmental and Epileptic Encephalopathy: A Distinct Retinal Phenotype in Human

**DOI:** 10.1111/cge.14724

**Published:** 2025-02-09

**Authors:** Oliver R. Marmoy, Eleanor Hay, Richard Bowman, Dorothy A. Thompson

**Affiliations:** ^1^ Clinical and Academic Department of Ophthalmology Great Ormond Street Hospital for Children London UK; ^2^ UCL‐GOS Institute of Child Health London UK; ^3^ Department of Clinical Genetics Great Ormond Street Hospital London UK; ^4^ International Centre for Eye Health, London School of Hygiene and Tropical Medicine London UK

**Keywords:** DNM1, dynamin, ERG, encephalopathy, epilepsy, retina, synapse, vision

## Abstract

Dynamin‐1 is an essential enzyme involved in the recycling of synaptic vesicles, in particular in the scission of endocytic buds within the pre‐synaptic terminal. Heterozygous pathogenic variants in *DNM1* result in Developmental and Epileptic Encephalopathy type 31A, where patients exhibit early onset refractory epilepsy, severe‐profound intellectual disability and poor visual behaviour. We present data demonstrating that this disorder progressively affects retinal synaptic function which, to our knowledge, is the first report of this phenotype in human. Clinical notes of the proband were reviewed incorporating ophthalmic phenotyping (imaging, electroretinography (ERG), pattern visual evoked potentials (PVEPs) and visual symptoms). Genetic testing was performed using trio whole genome sequencing. Genetic testing confirmed a *de‐novo* pathogenic variant in *DNM1*, a recurrent heterozygous missense variant, c.709C > T;p.(Arg237Trp). Serial ERG testing at 1, 3, 9 and 12 years old indicated a progressive inner retinal dysfunction affecting both rod and cone synaptic pathways mirroring the abnormalities in the mouse model of dnm1, with normal retinal structure. DNM1 affects retinal synaptic recycling and endocytosis and our findings show likely usefulness of ERG testing in affected individuals. Further work is needed to expand our understanding of how different *DNM1* variants affect retinal function.

## Graphical abstract

Variants in the *DNM1* gene cause a progressive cone‐rod synaptic dysfunction, evidenced by the electroretinogram. This is due to retinal synaptic abnormality due to poor endocytic scission of synaptic buds at the synapse consequent on Dynamin‐1 abnormalities.

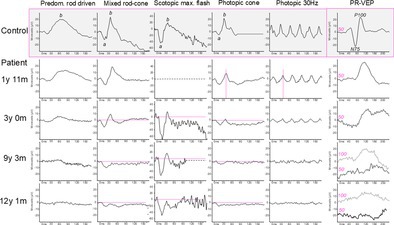

## 1 Introduction

Dynamin (DNM) is a superfamily of mechanochemical enzymes responsible for trafficking and recycling of synaptic vesicles, in particular the endocytic scission of clathrin‐coated vesicles [1]. Clathrin coated vesicles formed at the plasma membrane are subject to a scission through a fission reaction. This is achieved by Dynamin, which forms a helical polymer around the neck of neurotransmitter buds to make a conformational change to pinch off vesicles [2]. In mammals, there are three known dynamin isoforms, DNM1, DNM2 and DNM3 [3]. DNM1 is thought to be exclusively expressed in neural tissue, where it is predominantly located within presynaptic terminals [2]. Dynamin‐1 has been demonstrated to comprise of five major domains; a GTPase, a stalk region, a PH domain, a GED domain and PRD domain [4].

Pathogenic variants in the *DNM1* gene (*OMIM 602377*), located on the long arm of chromosome 9 at position 34.11, commonly affect coding of the GTPase domain [5]. These are most frequently due to *de novo* missense mutations, which, when heterozygous, result in autosomal dominant Developmental and Epileptic Encephalopathy, type 31A (DEE31A; OMIM 646346). This very rare disorder typically presents in infancy with global developmental delay, infantile onset severe refractory epilepsy, generalised hypotonia and severe to profound intellectual disability [5, 6, 7]. DEE31A typically results from *DNM1* variants that are predicted to have a dominant‐negative effect on the amino acid chain produced by *DNM1* [8, 9].

Visual abnormalities in *DNM1* are reported to be severe, often described as no or very poor visual fixation behaviour [10], and rarely may involve nystagmus [6]. Given the abnormal neurological basis of the disorder, these features have been suggested to be a result of cerebral visual impairment CVI [5, 6, 11]. However, it is known that *DNM1* is widely expressed in retinal tissue for endocytic recycling and therefore these visual symptoms may be attributable to abnormal synaptic signalling in the retina. Herewith, we report an individual with DEE31A demonstrating a progressive cone‐rod retinal synaptic dysfunction, which to our knowledge is the first ophthalmic report of this phenotype in human.

## 2 Methods

Written informed consent was obtained from the family of the patient. Electronic case‐notes were reviewed to characterise the ophthalmic and neurological phenotype. Panel based trio whole genome sequencing (WGS) of proband and parents was performed (PanelApp Paediatric disorders Version: 52.5).

Serial visual electrophysiology testing was performed, including pattern visual evoked potentials (PVEP) and flash electroretinograms (ERGs). Pattern VEPs were performed to a range of check widths incorporating ISCEV Standards [12], with flash ERGs performed according to a well‐established alternative paediatric protocol [13]. Fundus imaging was performed (Optos, Dunfermline, Scotland) alongside hand‐held optical coherence tomography (OCT) (Envisu C2300, Leica Microsystems, Germany).

## 3 Results

### 3.1 Clinical Presentation

We present a 12‐year‐old male of Sri Lankan heritage. The patient was born to non‐consanguineous parents, non‐dysmorphic and at the age of 3–4 months presented well grown (weight and head circumference on 98th centile) but with developmental delay, axial hypotonia, head lag and new‐onset seizures. His MRI was structurally normal and revealed only postural plagiocephaly. By the age of 8 months, significant developmental delay was apparent, with poor control of his epilepsy requiring multiple anti‐epileptic medications.

Ophthalmic review at the age of 21 months showed an intermittent fix and follow, but mostly inconsistent following of light or response to Cardiff acuity cards. His pupils were sluggish with no RAPD or Lisch nodules. By the age of 12 years the patient had a retinoscopic refraction of +3.50/−4.00 × 180 in each eye. The patient could fix and follow a 7 cm toy held at 20 cm. The patient's father perceived no deterioration of vision over time and reported that his son could pick up small objects and sometimes identify faces.

**FIGURE 1:**
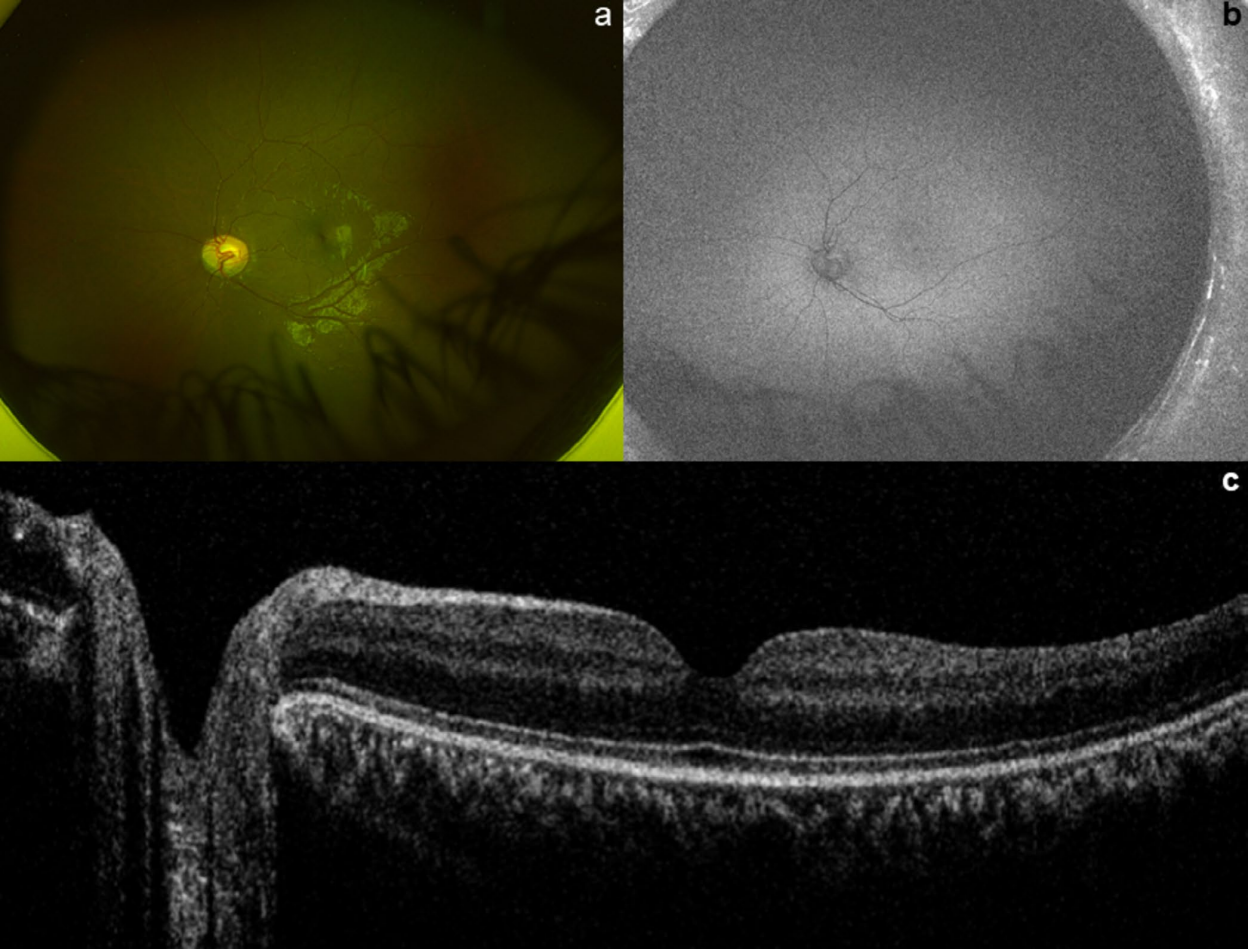
Retinal imaging. Panel a shows an OPTOS pseudocolour fundus image of the left eye, with panel b showing an OPTOS autofluorescence fundus image of the left eye without clear abnormalties. Panel c shows an OCT of the posterior pole illustrating appropriate macular structure.

Ultrawide pseudo‐colour and fundus autofluorescence imaging (Figure 1a,b) of the left eye did not demonstrate any significant retinal abnormalities, although the optic nerve had slight pallor. Handheld OCT of the macula (Figure 1c) showed appropriate retinal thickness and lamination, with no evidence of gross abnormality. It was not possible to image the right eye due to limited patient compliance, but this was noted to be similar to the left eye on clinical examination.

### 3.2 Genetic Testing

Trio WGS testing identified a *de novo* heterozygous pathogenic variant in *DNM1 (NM_004408.3)* in the proband, c.709C > T;p.(Arg237Trp). This recurrent missense variant has been reported multiple times in association with Developmental and Epileptic Encephalopathy type 31A (OMIM 616346).

### 3.3 Visual Electrophysiology

**FIGURE 2:**
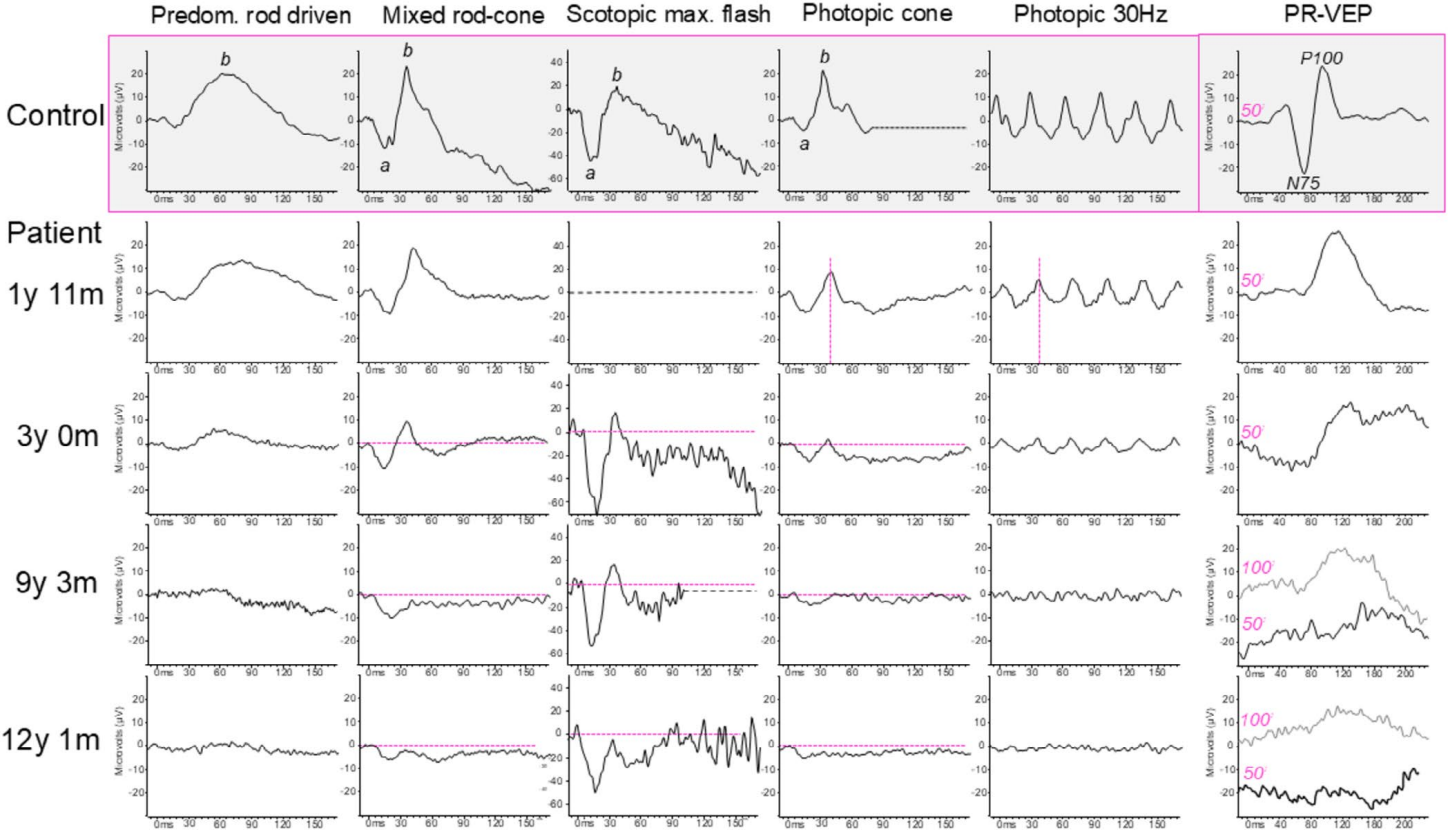
Electroretinogram and pattern reversal visual evoked potential (PR‐VEP) traces at each patient visit. An example control participant ERG array is provided across the top row (shaded). Each ERG step column is arranged according to age (left side). The PRVEP traces are shown according to ISCEV standard large check widths (50′) alongside to larger check widths (100′). [Colour figure can be viewed at wileyonlinelibrary.com]

Flash electroretinograms were performed on four occasions over a 10 year period (Figure 2). The ERG waveforms at 23 months first showed normal rod‐system function with mild cone system dysfunction evidenced by a delayed b‐wave and 30 Hz flicker peak‐time. This progressed by age 3 where generalised inner retinal dysfunction was evidenced by a subnoral rod‐driven b‐wave and reduction of cone mediated ERGs, producing a low b:a ratio. At the age of 9 years further deterioration was evidenced by the predominantly rod‐driven b‐wave becoming barely detectable, and further worsening in the cone ERG b:a ratio the b:a ratio with the 30 Hz flicker ERG equivocally evident above noise levels. By age 12 years there was global b‐wave amplitude reduction with a b:a ratio < 1 across ERGs, including the scotopic maximal flash ERG which was not a feature of earlier tests. The scotopic maximal flash a‐wave indices of rod photoreceptor function remained within reference range throughout visits until mildly reduced at 12 years of age (Figure S1). Pattern reversal VEPs (prVEPs) demonstrated progressive macular pathway dysfunction across visits (Figure 2).

## 4 Discussion

We present evidence that heterozygous *DNM1*‐related developmental and epileptic encephalopathy shows a progressive cone‐rod synaptic retinal dysfunction in childhood. Our patient presented with similar visual features to other reports in *DNM1* related disorders, comprising poor fixation, lack of following and poor visual behaviour. Existing reports have attributed poor visual behaviour to CVI [14], but these data suggest a progressive retinal synaptic abnormality, highlighting usefulness of a full ophthalmic review with an ERG.

Our patient showed early and subtle cone‐system dysfunction manifesting as mildly delayed cone b‐wave peak‐times. Thereafter, there was deterioration of the inner retinal function of both rod and cone system, which progressed rapidly between the ages 3–9 years and continued until the most recent test age 12 years. The scotopic a‐wave indices of photoreceptor function remained within reference limits, although had reduced in amplitude mildly across visits (Figure S1) suggesting there may be some photoreceptor involvement later in life.

The significance of the retinal abnormalities are challenging to contextualise in view of the patient's accompanying communication and intellectual difficulties which limited formal acuity testing. Certainly, whilst the patient had an early and relatively rapid deterioration in visual function, thereafter his father perceived that vision was stable. The extent of pattern VEP deterioration appeared less marked than seen for the ERG, which would suggest some relative preservation of macular pathway integrity. Interestingly, the patient's visual symptoms did not clearly relate to the chronological ERG changes, suggesting CVI is likely a comorbidity in this condition as previously reported.

To our knowledge, this is the first report of this ocular phenotype in *DNM1* associated disorders. We could locate only two existing cases which report limited detail of retinal abnormalities. The first is a patient with an autosomal recessive (AR) *DNM1‐*related disorder [15], reported to have a ‘moderate rod‐cone dystrophy’, in a although no ERG data are described. This patient was also remarked to have nystagmus, which is an uncommon feature of a primary rod‐cone dystrophy and is more typical of cone dystrophies or inner retinal dysfunction, suggesting the exact retinal abnormality is ambiguous. The second case also had AR *DNM1*‐related disorder and limited ERG data [16]. This patient was diagnosed with an early‐onset retinal dystrophy (EORD) based on a severely reduced amplitude mixed cone‐rod ERG, but the authors also report an impaired b:a ratio showing a trend for an electronegative waveform. Typically, EORD involves severely reduced a‐wave amplitude, often with relative preservation of the b:a ratio (if evident), though this can be reduced. Their findings report ERG data only to mixed cone‐rod and photopic 30 Hz stimuli, but we may infer from the reported reduced b:a ratio that this patient may also exhibit a similar retinal synaptic dysfunction to our case. Nonetheless, our case is the first to demonstrate a progressive inner retinal synaptic dysfunction resulting in an electronegative ERG supported by serial electrophysiological data.

The progressive inner retinal abnormality exhibited with age is supported by the pathogenesis of *DNM1* variants. Retinal photoreceptors are highly energy dependent and therefore have high requirements for vesicular recycling and transport [17]. As Dynamin‐1 is expressed across multiple parts of the retina including the rod‐ribbon synapse, this abnormality has potential to affect synaptic recycling and transmission at the photoreceptor‐bipolar cell synapse [18]. Mice with *dnm1* mutations demonstrate functional neural synapses at birth, but as the disease progresses, tubular membrane invaginations along with clathrin‐coated buds accumulate at the synapse from incomplete membrane scission, which reduces its functional integrity [19]. Most interestingly, the effect of Dynamin‐1 mutations on retinal function has recently been reported in mice with *dnm1* mutations, demonstrating that the scotopic ERG shows early b‐wave amplitude reduction which is progressive with later mild a‐wave reduction. This is almost identical to the features observed in our case [20]. These data support that the detailed mouse data on *dnm1* functional effect in retina shares a very similar progressive ophthalmic phenotype in human.

Unfortunately, this would suggest that these functional changes are irreversible once acquired [19] and mice also show degeneration of bipolar cell dendrites following synaptic dysfunction [20]. Therefore, this advocates that early intervention is imperative to minimise the abnormal structural alterations within the retinal pre‐synaptic membrane. Recent developments in RNAi gene therapy and molecular treatment which have demonstrated acceleration of endocytosis in mice harbouring *dnm1* mutations, offer potential avenues for clinical treatments and outcome monitoring [21, 22, 23].

## 5 Conclusion

We report a patient with *DNM1‐*related developmental and epileptic encephalography associated with a progressive retinal cone‐rod synaptic dysfunction. The phenotype of this patient indicates that the visual symptoms reported in *DNM1‐*related DEE may arise at a retinal level, albeit co‐existent with CVI. These data suggest that other disorders of synaptic vesicle cycling may benefit from electroretinographic assessment.

## Author Contributions

Conceptualization: O.R.M. Data collection and analysis: O.R.M. and D.A.T. Data curation: O.R.M. and D.A.T. Investigation: All authors. Writing – original draft: O.R.M. Writing – review and editing: All authors.

## Consent

Written informed consent was obtained.

## Conflicts of Interest

The authors declare no conflicts of interest.

## Peer Review

The peer review history for this article is available at https://www.webofscience.com/api/gateway/wos/peer‐review/10.1111/cge.14724.

## Supporting information


Figure S1.

## Data Availability

The data that support the findings of this study are available from the corresponding author upon reasonable request.
