# Supplementary material for: Progressive Cone‐Rod Synaptic Dysfunction in Dynamin‐1 ( DNM1 ) Related Developmental and Epileptic Encephalopathy: A Distinct Retinal Phenotype in Human
Source: Clin Genet. 2025 Feb 9;108(2):194–8. doi: 10.1111/cge.14724 (PMC12215225; doi:10.1111/cge.14724)
Supplement: Supplementary file 1 — Figure S1. [file CGE-108-194-s001.docx]

**Supplemental Figure 1**


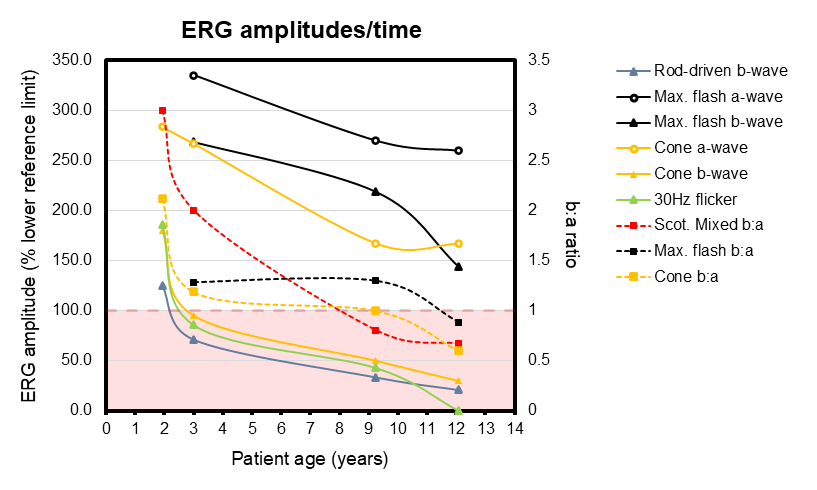


**Electroretinogram amplitudes plotted across patient visits according to age.** ERGs are coloured according to stimulus type; predominantly rod-driven (blue), scotopic maximal flash (black), photopic cone (orange), photopic 30Hz flicker (green) and scotopic mixed rod-cone (red). Raw a-wave amplitudes are plotted with open circle markers and raw b-wave amplitudes plotted with triangle markers, corresponding to the left Y-axis plotted according to % of relative lower reference limits. Square markers with dashed lines show the b:a ratio for each stimulus, corresponding to the right Y-axis. The shaded red area corresponds to the lower reference limit (left Y-axis) from the laboratory reference values (>120 participants) for which any value below this threshold would be considered abnormal.
